# A systematic and comprehensive analysis of T cell exhaustion related to therapy in lung adenocarcinoma tumor microenvironment

**DOI:** 10.3389/fphar.2023.1126916

**Published:** 2023-02-06

**Authors:** Peipei Hu, Jiahao Ma, Jinjian Chen

**Affiliations:** ^1^ Department of General Medicine, First Affiliated Hospital of Wenzhou Medical University, Wenzhou, China; ^2^ Key Laboratory of Nano-carbon Modified Film Technology of Henan Province, Diagnostic Laboratory of Animal Diseases, School of Pharmacy, Xinxiang University, Xinxiang, China

**Keywords:** T exhausted, lung adenocarcinoma, single cell sequencing, biomarkers, tumor microenvironment

## Abstract

**Background**: T cell exhaustion (TEX) is an important immune escape mechanism, and an in-depth understanding of it can help improve cancer immunotherapy. However, the prognostic role of TEX in malignant lung adenocarcinoma (LUAD) remains unclear.

**Methods**: Through TCGA and GEO datasets, we enrolled a total of 498 LUAD patients. The patients in TCGA-LUAD were unsupervised clustered into four clusters according to TEX signaling pathway. WGCNA analysis, survival random forest analysis and lasso regression analysis were used to select five differentially expressed genes among different clusters to construct a TEX risk model. The risk model was subsequently validated with GEO31210. By analyzing signaling pathways, immune cells and immune checkpoints using GSEA, GSVA and Cibersortx, the relationship between TEX risk score and these variables was evaluated. In addition, we further analyzed the expression of *CCL20* at the level of single-cell RNA-seq and verified it in cell experiments.

**Results**: According to TEX signaling pathway, people with better prognosis can be distinguished. The risk model constructed by *CD109, CCL20, DKK1, TNS4*, *and TRIM29* genes could further accurately identify the population with poor prognosis. Subsequently, it was found that dendritic cells, *CD44* and risk score were closely related. The final single-cell sequencing suggested that *CCL2O* is a potential therapeutic target of TEX, and the interaction between TEX and CD8 + T is closely related.

**Conclusion**: The classification of T cell depletion plays a crucial role in the clinical decision-making of lung adenocarcinoma and needs to be further deepened.

## 1 Introduction

The mortality rate of lung adenocarcinoma remains high throughout the world (Relli et al., 2019). LUAD is the most common form of primary lung cancer. Smoking-primary or secondary exposure, are the main causes (Hutchinson et al., 2019). The traditional treatment of LUAD includes surgical resection, chemotherapy, and radiotherapy. A number of new therapeutic approaches have also been discovered that can be used to treat LUAD, such as immunotherapy (Succony et al., 2021).

As a subset of T immunocytes, CD8^+^ T lymphocytes are responsible for mediating the activity of the T immunocytes to chronic infections and cancers (Zhang and Bevan, 2011). Chronic infections and tumor antigens cause differentiated CD8 + T cells to exhaust (Speiser et al., 2014; McLane et al., 2019). The expression of cytokine suppression, decreased killing, and hypoproliferation of T cells are all symptoms of T cell exhaustion (TEX), which occurs as a result of these processes (Freeman et al., 2006; Zhang et al., 2022). The immune checkpoint inhibitors working mechanism is not depleting T cells in the immune microenvironment, and TEX is thought to be a pathway of resistance (Chow et al., 2022). In parallel, Immunotherapy to restore TEX responses has transformed the current clinical decision for cancer treatment (Hudson and Wieland, 2022). There has been evidence that inhibiting the PD-1 inhibitory receptor pathway can reactivate the TEX response and active the immune anti-tumor effect (McLane et al., 2019).

Although T lymphocytes in the body can attack tumors, the latter often present a highly reactive microenvironment that shuts down the killing capacity of T cells (Ma et al., 2019). The tumor microenvironment (TME) is a key factor in the escape of tumor cells from the immune system, and this environment plays a key role in cancer development (Gholami et al., 2017). In the TME, T cells are regulated by a complex immunosuppressive network consisting of cancer cells, inflammatory cells, stromal cells and cytokines (Jiang et al., 2015). Among these TME components, cancer cells, inflammatory cells, and suppressor cytokines have key roles in regulating T cell phenotype and function (Speiser et al., 2016). These components contribute to the eventual differentiation of T cells into “exhausted” T cells. Eventually, the majority of T cells in the TME differentiate into exhausted T cells that express high levels of suppressor receptors, produce fewer effector cytokines, and lose the ability to eliminate cancer.

In the initial characterization of exhausted T cells, the levels of transcription factors T cell factor (TCF1) and programed cell death protein (PD-1) expression were used to distinguish between the progenitor and terminally differentiated subtypes (Im et al., 2016; Siddiqui et al., 2019). As a result of progenitor exhaustion, T cells exhibit stem cell characteristics or memory characteristics, which enable them to self-renew and transform into terminally differentiated cells (Akbar and Henson, 2011; Utzschneider et al., 2016). Comparatively, the terminally differentiated branching subtype does not have a functional recovery potential and is limited in its expansion potential (Philip et al., 2017; Khan et al., 2019). In another study, TEX was divided into four stages based on Ly108 and CD69 expression (TEX^prog1^: Ly108 + CD69^+^; TEX^prog2^: Ly108 + CD69^−^; TEX^int^: Ly108-CD69^−^; TEX^term^: Ly108-CD69^+^) (Beltra et al., 2020). These studies have shown the TEX process is dynamic, with a phenotypic and functional continuum of intermediate states, indicating a developmental hierarchy (Zhang et al., 2022). Further researches showed that individual patients displayed different levels of T cell exhaustion (Kim et al., 2021) and the presence of T cell activation or exhaustion biomarkers such as sTIM-3, CD25 in patients is evidence of this, these markers are associated with a poor outcome (Berg et al., 2022). In a pan-cancer analysis, Zhang et al. obtained TEX-related genes through machine learning to classify tumors in different things TEX for clinical decision-making (Zhang et al., 2022).

In this study, we performed clustering analysis on the TCGA-LUAD data through TEX-related pathways, and further WGCNA and random survival forest and lasso regression analyses to construct TEX risk scores. Subsequently, the relationships between TEX risk scores and GSEA pathway enrichment analysis, GSVA pathway enrichment, and CIBERSORTX immune infiltrating cells were analyzed. 348 urothelial cancer patients which treated with atezolizumab (PD-L1) were collected to examined the effect of TEX risk score on immunotherapy effectiveness. Single-cell sequencing data and experiment were finally used to analyze potential therapeutic targets and cell communication in TEX.

## 2 Materials and methods

### 2.1 Data acquisition

**FIGURE 1:**
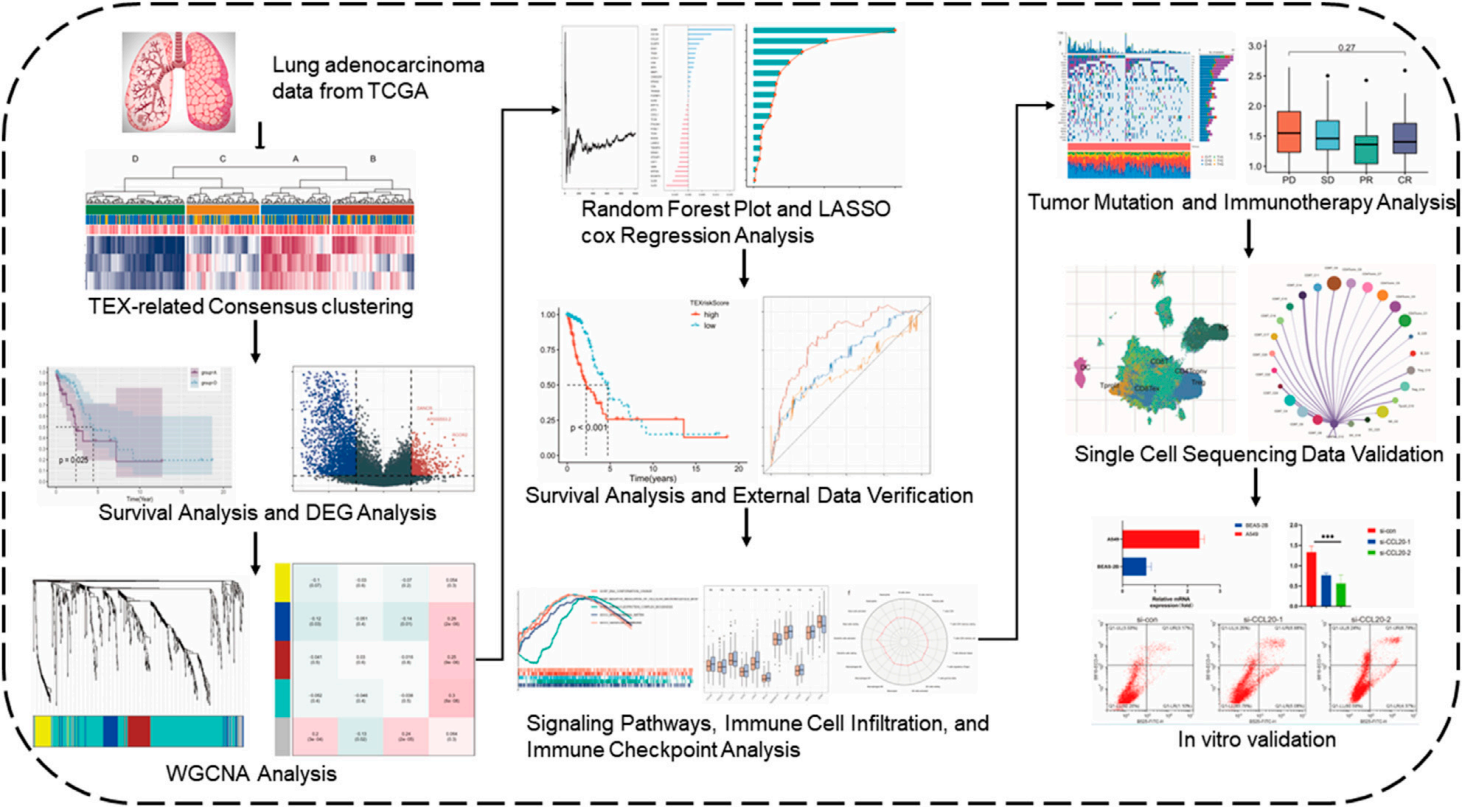
Flow chart of the study.

The expression data, gene mutations, and clinical information were collected from the Cancer Genome Atlas (TCGA) website for 284 patients with LUAD (Tomczak et al., 2015). And 214 LUAD patients’ information were collected through dataset GSE31210 in the GEO database. Single-cell sequencing data (GSE176021) of tumor-infiltrating T lymphocytes from six NSCLC patients were obtained from the GEO database. For data inclusion criteria, we selected patient samples with RNA transcriptome sequencing data and complete clinical data. For data from different datasets de-batching effects were performed and normalized, and we used fragment per kilobase transcript/fragment per million mapping (FPKM) expression values for further analysis. In FPKM, RNA-seq data were normalized to the length of each gene and the total number of aligned reads in the library (Trapnell et al., 2010). FPKM values were transformed using log2 (FPKM + 1). The flowchart of our investigation was displayed in Figure 1.

### 2.2 Unsupervised cluster analysis

The molecular signature database provided information on TEX signaling pathways and marker genes (Wherry, 2011; Liberzon et al., 2015). Similar to previous studies, we performed an unsupervised cluster analysis of LUAD patients using IFN-γ, TNF, and IL-2 signaling pathways to represent the TEX pathway (Zhang et al., 2022). The specific method is to use ssGSEA through the “GSVA” R package to estimate the activity score of each patient’s TEX pathway (Hänzelmann et al., 2013). The percentage of patients at different stages in different clusters is also shown.

### 2.3 Comparison of overall survival between different clusters

To further explore the differences among different subgroups, we first drew Kaplan-Meier (K-M) survival curves for different clusters with the mark of 50% survival rate. Then the K-M survival curve of pairwise subgroups was drawn.

### 2.4 CIBERSORTX

To further explore the abundance of immunocytes in different classifications, we used CIBERSORTX algorithm to evaluate 22 immunocytes in samples from different clusters (Steen et al., 2020). After cell infiltration of each sample was obtained, COX regression analysis was conducted to explore the prognostic value of various cells in each TEX cluster. Based on median immune cell content, LUAD was divided into diverse subgroups, and survival rates were compared between groups.

### 2.5 Weighted gene co-expression network analysis

The correlation patterns among genomics can be described using a systems biology method known as a weighted correlation network analysis (WGCNA) (Langfelder and Horvath, 2008). The R package repository had the package WGCNA 3.6.1 that was used for the WGCNA. WGCNA analysis was performed after deunion of the four cluster differential genes. The significance of each gene was taken into account when calculating the association between the gene expression profile and the TEX score, and the relationship between module eigengenes and gene expression profiles was taken into consideration when determining module membership. The soft threshold parameters were set at a power of 4 and a scale-free R2 of 0.9, in order to ensure the topology network was scale-free despite the number of nodes. The analysis consisted of retrieving an initial set of six modules, and the Grey modules that showed the strongest correlations were applied for further investigation.

### 2.6 RandomForest

Using the survival random forest of 1,000 trees by the R package randomSurvivalForest version 3.6.4, it was possible to validate the results and rank the importance of 7 genes obtained from Lasso regression using the R package randomSurvivalForest Version 3.6.4 (Taylor, 2011). The relative importance of gene > 0.2 is considered the ultimate hub gene.

### 2.7 Construction and validation of risk models

Gene expression tends to show significant collinearity between genes, which means it is necessary to use prognostic models if needed. LASSO regression and other methods reduce the number of variables to further reduce the redundancy of the model and increase the convenience of clinical use. Based on the following formula, we were able to calculate the risk score according to the following (Tibshirani, 1997):

$$Risk\;score=\overset{n}{\underset{i=1}{\sum }}{\mathrm{Exp}}_{i}*{Coef}_{i}$$

Here, the TCGA-LUAD data set was employed as the training set to construct the risk model of LUAD patients based on survival random forest screening genes, took OS as the outcome event, and *p* value less than 0.05 as the limit of statistical significance. We then categorized the patients into diverse subgroups based on the formula generated by the risk model. Meanwhile, the K-M survival curve was drawn for the high and low risk group. Receiver operating characteristic (ROC) curves have a wide range of uses in identifying the diagnostic power of threshold changes. To further analyze the predictive power of prognostic models, we plotted the ROC. An analysis of multivariate cox regression was undertaken in order to determine the independent prognostic significance of risk scores. To ensure the value of external generalization of the prognostic model, we used another LUAD dataset (GSE31210) for validation.

### 2.8 Biological function in relation to risk score

A follow-up analysis looked at genes with differential expression (DEGs) between high and low risk groups. It was established that the cut-off criteria for the study were |FC| > 2 and adj. p.val > 0.05. GSEA software was used to analyze three data sets of HALMARK, KEGG and GO for the biological functional differences among high-risk patients (Shi and Walker, 2007; Subramanian et al., 2007).

### 2.9 GSVA analysis between high-And low-risk groups

By using the GSVA analysis, it was possible to explore the differences between subgroups in signaling pathways for disease development (Hänzelmann et al., 2013). Moreover, a correlation analysis was conducted between the partial signal pathway score and the risk score.

### 2.10 The predictive significance of TEX risk model

A boxplot was made to illustrate the expression of 11 immune checkpoints between different subgroups. An analysis of 22 different types of immune cell infiltration was performed using the CIBERSORTX algorithm.

The detailed gene mutation statuses of the subgroups were displayed using the R package “maftools” to make comparisons between the two subgroups (Mayakonda et al., 2018). With the IMvigor210 package, we were able to determine gene expression and immunotherapeutic effectiveness in the IMvigor210 cohort (Mariathasan et al., 2018). IMvigor210 cohort is widely used to analyze the efficacy of immunotherapy.

### 2.11 Single cell sequencing analysis

It is a standard processing procedure that is used to do downstream processing on scRNA-seq data which is carried out using Seurat R software package, version 3.0.2, and a standard downstream processing package for this analysis (Stuart et al., 2019). In addition, genes detectable in fewer than 3 cells and genes detected in fewer than 200 cells were excluded, and the percentage of mitochondria detected was limited to no more than 20% of the total number of genes. Then, t Data was normalized using LogNormalize. A non-linear method used for reducing the dimensions of a sample is t-distributed stochastic neighborhood embedding (t-SNE) that is used for unsupervised clustering and unbiased visualization of cell populations on a two-dimensional map after principal component analysis (PCA) (Van Der Maaten and Hinton, 2008). A minimum fraction of 0.25 cell population fraction was used in both populations in order to identify marker genes in each cluster using the “FindAllMarkers” function. The filtering criterion was filter value of absolute log2 fold change (FC) ≥1. To visualize each marker gene’s expression patterns within the cluster, the “DotPlot” function in Seurat was used. Then, the SingleR package (version 1.0.0) was utilized for annotating cell types based on marker-based information (Aran et al., 2019).

### 2.12 Cell culture

The A549 and BEAS-2B cell lines were obtained from Dr Liu. A549 cell lines were cultured in RPMI‐1640 (Invitrogen) and BEAS-2B cells were cultured in DMEM medium, The medium was supplemented with 10% FBS (Gibco).

### 2.13 Molecular expression verification

The expression of *CCL20* in tumor and normal tissues of LUAD patients was compared through GEPIA2 online website, and we analyzed the overall survival rate of high expression group and low expression group (Tang et al., 2017).

The total RNA was extracted using the Trizol reagent. RT was performed with DNA-free total RNA in Revert Aid First Strand cDNA Synthesis Kit (Thermo). For PCR amplification, specific primers were used to amplify the transcribed cDNA. *CCL20* Forward: ATG​TGC​TGT​ACC​AAG​AGT​TTG​C; *CCL20* Reverse: CCA​ATT​CCA​TTC​CAG​AAA​AGC​C.

Integrated DNA technologies (Coralville, IA, United States) provided us the synthetic siRNA and the scrambled negative control siRNA. This experiment consisted of transfecting cells with LipofectamineTM RNAiMAX (Thermo Fisher Scientific, Massachusetts, United States) in opti-MEM according to the procedure given by the manufacturer.

### 2.14 Flow cytometry

The manufacturer’s instructions were followed when performing flow cytometry. Apoptosis was detected with the Annexin V-PE/7-ADD Apoptosis Detection Kit (Vazyme, A213-01). The B525 nm wavelength was selected for the Fluorescein (FITC) signal channel, and the B610 nm signal channel was selected for the ECDPE-TR (ECD) signal channel.

### 2.15 Statistical analysis

It was determined that two groups with normally distributed variables and those with variables that were not normally distributed were statistically significant using independent t-tests and Mann-Whitney U tests. In order to make a comparison between the two groups on the basis of differences between the groups, we conducted an analysis of variance (ANOVA) and a Kruskal–Wallis test (Hazra and Gogtay, 2016). We performed Spearman correlation and distance correlation analyses using the R package Hmisc 4.4.1. To analyze the correlation between the objects. Those objects whose coefficient was greater than 0.5 were considered highly correlated (Faul et al., 2007). For the purpose of identifying the prognostic factors, Cox regression analyses were conducted. A survival curve with all survivorship curves generated by the R package survminer was also used to determine the overall survival (OS) and TEX riskScore values before generating any survival curves with the R package survminer. As a means of plotting the heatmaps, the R package Complex Heatmap 2.4.3 was used. R package ggplot2 was used for visualizing data comparisons. There were two-sided statistical analyses conducted using R software, which was used for all statistical analyses.

## 3 Results

### 3.1 Unsupervised cluster analysis

**FIGURE 2:**
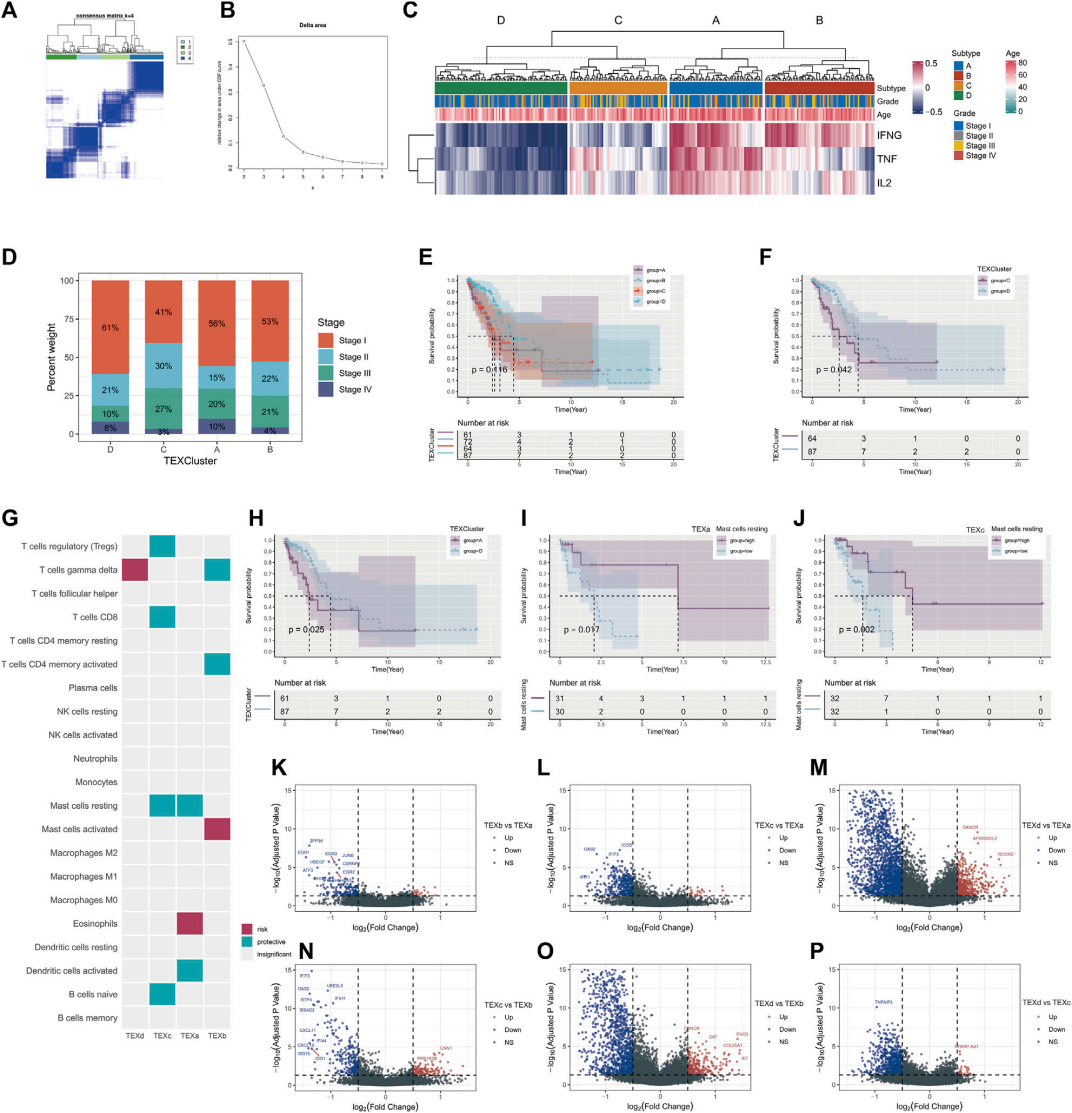
Unsupervised cluster analysis was performed on LUAD patients according to TEX signaling pathway. Unsupervised cluster analysis of patients with TCGA□LUAD (consensus matrix k = 4) **(A)**. Delta area of unsupervised consensus cluster analysis **(B)**. Heatmap of scores for four T cell exhaustion and IFNG/TNF/IL-2 signaling pathways **(C)**. Percentages of different clinical stages in TEX clusters **(D)**. Kaplan-Meier survival curves for the four TEX clusters **(E)**. Kaplan-Meier survival curves for TEX C and TEX D **(F)**. Kaplan-Meier survival curves for TEX A and TEX D **(H)**. The cibersortX algorithm in four TEX clusters was used to analyze the infiltration results of 22 immune cells **(G)**. K-M survival curves of patients with high and low abundance of mast cells resting in TEXa **(I)** and TEXc **(J)**. Volcano plot of differential genes between TEXa and TEXb **(K)**, TEXc and TEXa **(L)**, TEXd and TEXa **(M)**, TEXc and TEXb **(N)**, TEXd and TEXb **(O)**, TEXd and TEXc **(P)**.

The outcome of unsupervised cluster analysis were displayed in Figures 2A, B, where the best result was to classify the 284 LUAD patients into four TEX cluster (consensus matrix k = 4) according to the GSVA scores of the three IFNG/TNFA/IL-2 pathways. Figure 2C displayed the GSVA scores of the three pathways within the four clusters. Cluster B has the highest IFNG/TNF/IL-1 pathway score, and cluster D has the lowest score. The number of patients with stage1 in cluster b was larger than in cluster A, B and C. The number of patients with stage1 in cluster B was larger than that in cluster A, B and C (Figure 2D), suggesting that the overall survival of patients in cluster b may be better than that in other cluster populations. We plotted K-M survival curves for the overall survival of the four cluster populations, and there was no statistically significant difference (*p* = 0.116) in survival between the four clusters (Figure 2E). We plotted the K-M survival curves separately for cluster B and cluster A C D, and the results suggested that the overall survival rate of cluster B was higher than that of cluster A C D (Figures 2F-H; Supplementary Figure S1) (Cluster C vs. D, *p* = 0.042; cluster A vs. D, *p* = 0.025; cluster B vs. D, *p* = 0.073).

To further analyze the abundance of immune cells in different cluster, we applied the CIBERSORTX algorithm to evaluate the 22 immune cells in the samples in different clusters. After cell infiltration score was obtained for each sample, COX regression analysis was performed to explore the prognostic value of various cells in each TEX cluster. Mast cell resting was a protective factor in both TEXa and TEXc (Figures 2I, J).

For further analysis of the transcriptome differences between different cluster, we will contrast between different cluster differences in gene analysis, analysis of the standard is greater than or equal to | logFC | = 0.5, rectify the *p* value is less than 0.05, and mapped the volcano map is used to display the results of the analysis (Pearson correlation coefficient = 0.2, *p* value < 0.001) (Figures 2K–P).

### 3.2 WGCNA and survival random forest results

**FIGURE 3:**
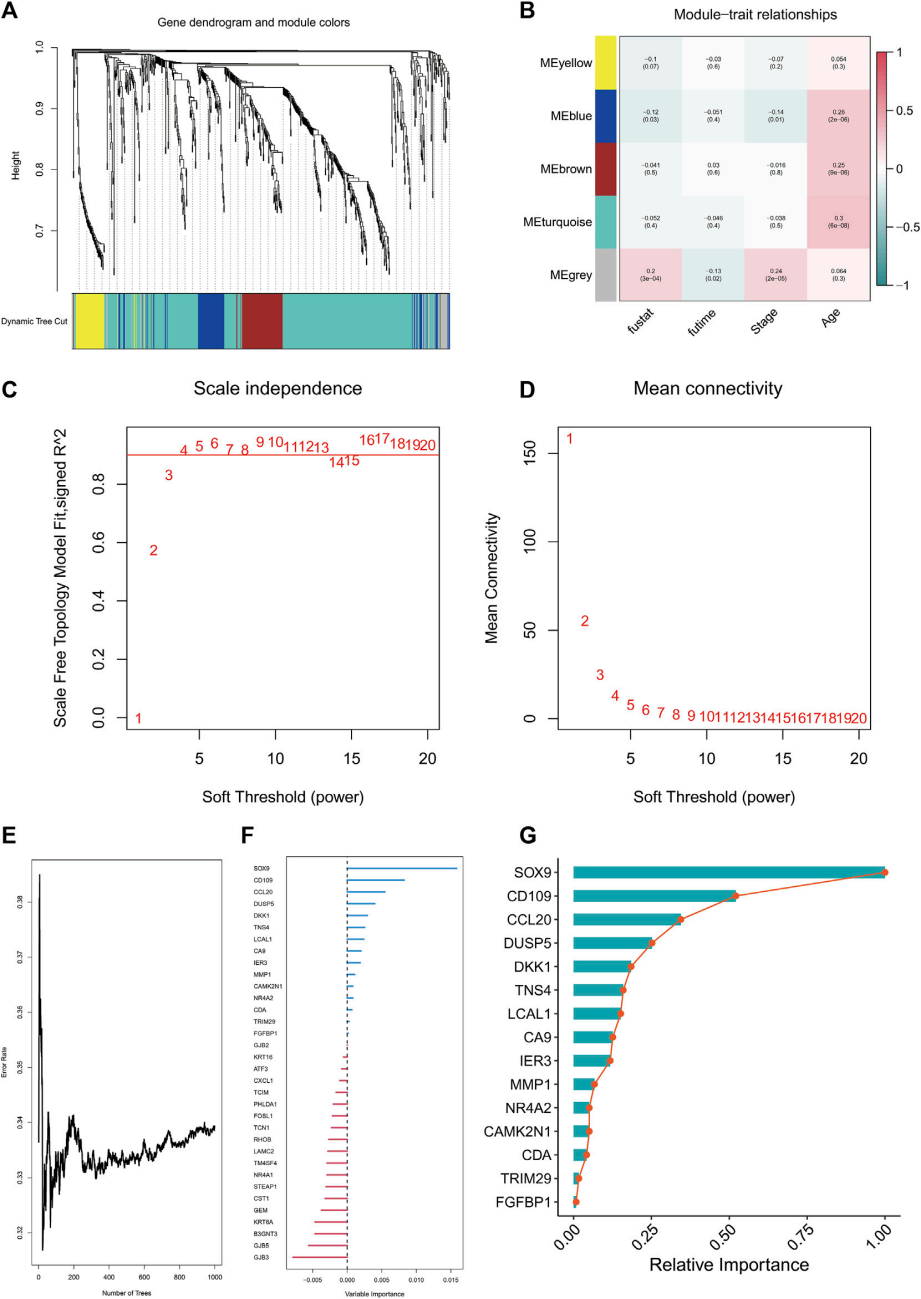
WGCNA analysis and random survival analysis. Clustering dendrogram of TCGA-LUAD **(A)**. Heatmap of correlation between WGCNA modules and clinical features **(B)**. Various soft thresholding powers are calculated according to their scale-free fit index **(C)**. Soft-threshold power mean connectivity analysis **(D)**. Plot of random survival forest based on number of trees and error rate **(E)**. Variable Importance ranking of genes in random survival forests **(F)**. Relative Importanc ranking of genes in random survival forests **(G)**.

WGCNA analysis results suggested that the grey module was most relevant to survivals related information, and the grey module was selected for subsequent analysis (Figures 3A–D). There were 36 genes chosen as hub genes in the Grey module since they had absolute values of module membership [MM] that were greater than 0.5 and absolute values of gene significance [GS] that were greater than 0.5 within the module (Supplementary Table S1). Variable selection based on minimum depth values above the threshold (0.001) and importance values above the threshold (0.2) yielded seven tentative (*SOX9*, *CD109*, *CCL20*, *DUSP5*, *DKK1*, *TNS4*, *and LCAL1*) candidate prognostic markers for LUAD. This suggests that these seven genes are most relevant to the prognosis of LUAD (Figures 3E–G).

### 3.3 Development and validation of TEX risk model

**FIGURE 4:**
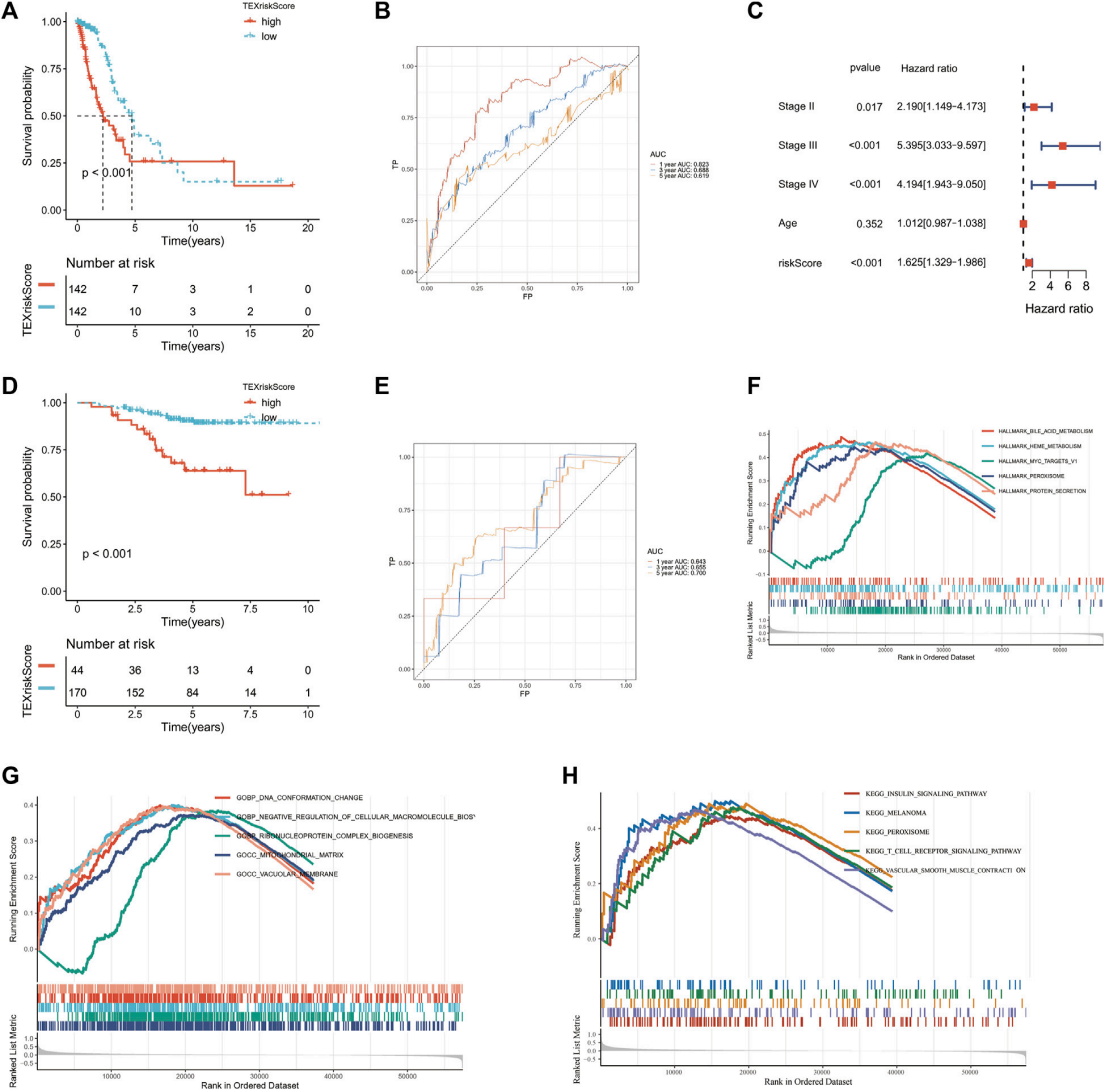
Construction and validation of TEX risk model. Kaplan-Meier (K–M) survival curves of patients in the high and low risk groups in TCGA-LUAD **(A)** and GSE31210 **(D)**. Multivariate Cox analysis in TCGA-LUAD cohort **(C)**. TEX risk model AUC values at year 1, 3, and 5 in TCGA-LUAD **(B)** and GSE32120 **(E)**. HALLMARK pathway enrichment analysis **(F)**, GO pathway enrichment analysis **(G)** and KEGG pathway enrichment analysis **(H)** of the high-risk group in TCGA-LUAD.

A TEX risk model that includes five genes was constructed using lasso regression analysis. The formula for the risk score is as follows: risk socre = (0.2628*CD109 + 0.0464*CCL20 + 0.0163*DKK1 + 0.0359*TNS4 + 0.0348*TRIM29). The TCGA-LUAD patients were divided into high- and low-risk groups based on their risk scores. The K-M survival curve between high and low risk groups suggested that the high-risk group had worse overall survival (*p* < 0.001) (Figure 4A). The AUC values of the TEX risk model were 0.823 in the first year, 0.688 in the third year, and 0.619 in the fifth year (Figure 4B). Multivariate COX analysis showed that TEX Score was an independent prognostic factor (*p* < 0.05, Hazard Ratio :1.625 [1.329−1.986]) (Figure 4C). In the validation set GSE31210, we also found that high-risk LUAD patients had worse OS (Figure 4D). The AUC values of TEX risk model in the validation set were 0.643 in the first year, 0.655 in the third year, and 0.700 in the fifth year. These results TEX risk model have good predictive power (Figure 4E).

### 3.4 TEX risk score and biological function GSEA analysis

Based on the HALLMARK, KEGG, and GO datasets, we performed an enrichment analysis of biological functions in high-risk patients using GSEA software. The results showed that the five HALLMARK pathways with the highest enrichment were bile acid metabolism, heme metabolism, MYC target v1, peroxisome, and protein secretion (Figure 4F). The five most enriched pathways in the GO database were DNA conformational changes, negative regulation of cellular macromolecular biosynthetic processes, ribonucleoprotein complex biogenesis, mitochondrial matrix, and vacuolar membrane (Figure 4G). The top five enriched KEGG pathways were insulin signaling pathway, melanoma, peroxisome, T cell receptor signaling pathway, and vascular smooth muscle contraction (Figure 4H). The results showed that high-risk patients were highly associated with many tumor proliferation and metabolism-related pathways, suggesting that targeted TEX affects the prognosis of LUAD patients mainly through tumor proliferation and metabolic pathways.

### 3.5 TEX risk score and GSVA analysis

**FIGURE 5:**
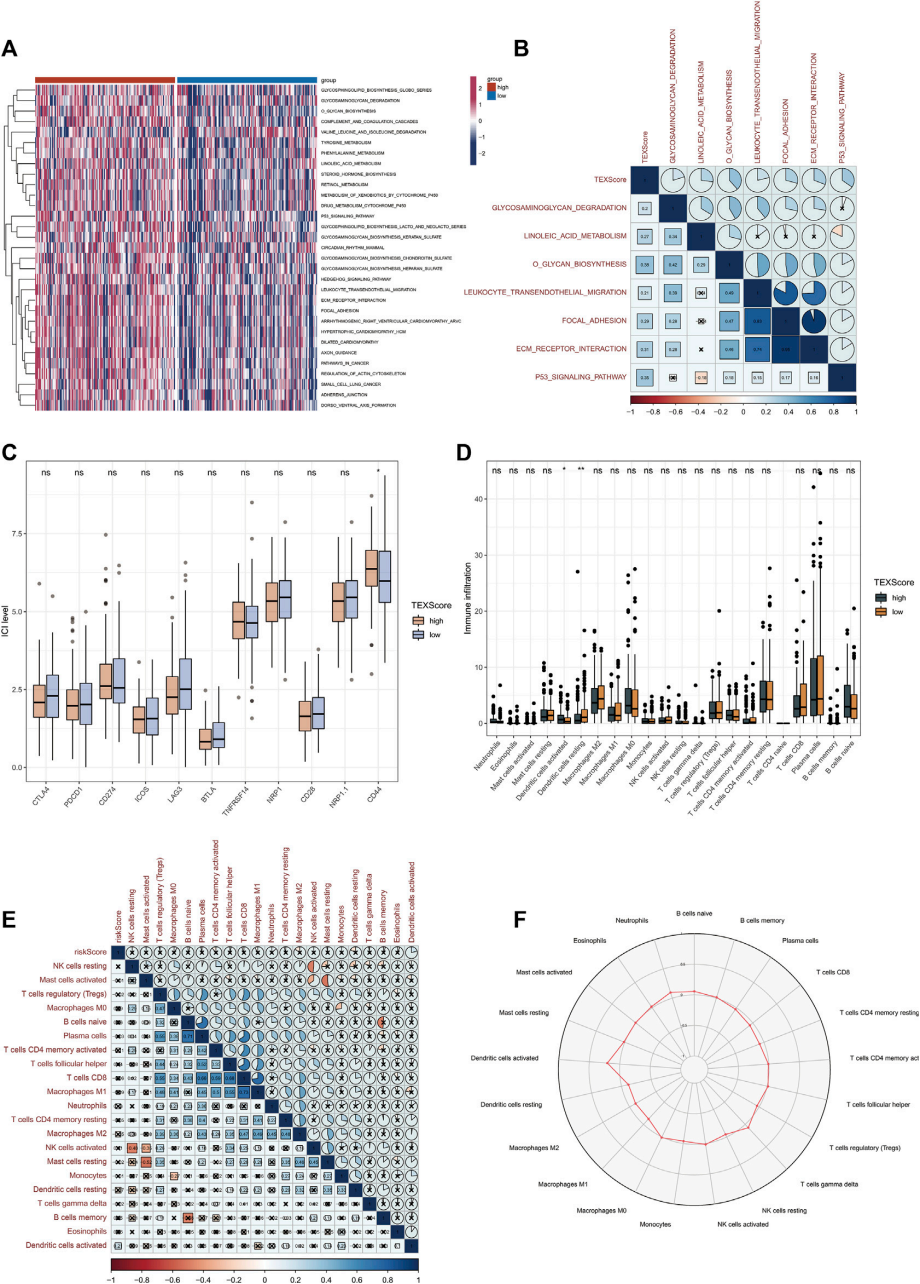
Relationship between TEX risk scores and immunity. GSVA analysis between high and low risk groups in TCGA-LUADA **(A)**. Correlation scores of TEX risk scores and signaling pathways **(B)**. Expression of 11 immune checkpoints in the high and low risk groups **(C)**. The abundance of 22 immune cells in cibersortx high and low risk groups was analyzed **(D)**. Association of TEX risk scores and 22 immune cells **(E)**. Radar plot of the abundance of 22 immune cells in the high-risk group **(F)**.

We selected several gene sets for GSVA analysis based on the above GSEA results and found that TEX score was positively correlated with glycosaminoglycan degradation, linoleic acid metabolism, o glycan biosynthesis, leukocyte transendothelial migration, focal adhesion, ECM receptor interaction and p53 signaling pathway (Figures 5A, B). This suggests a potential pathway through which TEX exerts its effects.

### 3.6 Relationship between TEX risk score and immunity

Considering the great potential of TEX for immunotherapy, In both high and low risk groups, we plotted the expression levels of 11 immune checkpoints. In the high-risk group, CD44 expression was higher, which may be a therapeutic target in the future (Figure 5C). Based on 22 immune cell infiltrations, the high-risk group had a higher percentage of resting Dendritic cells and a lower percentage of activated Dendritic cells (Figure 5D). Further correlations of TEX risk scores and 22 immunocytes are shown in Figure 5E. The radar chart further showed the contents of 22 immunocytes in the high-risk group (Figure 5F), suggesting that TEX may affect the prognosis of LUAD by regulating the state of Dendritic cells.

### 3.7 Relationship between TEX risk score and genetic mutations

**FIGURE 6:**
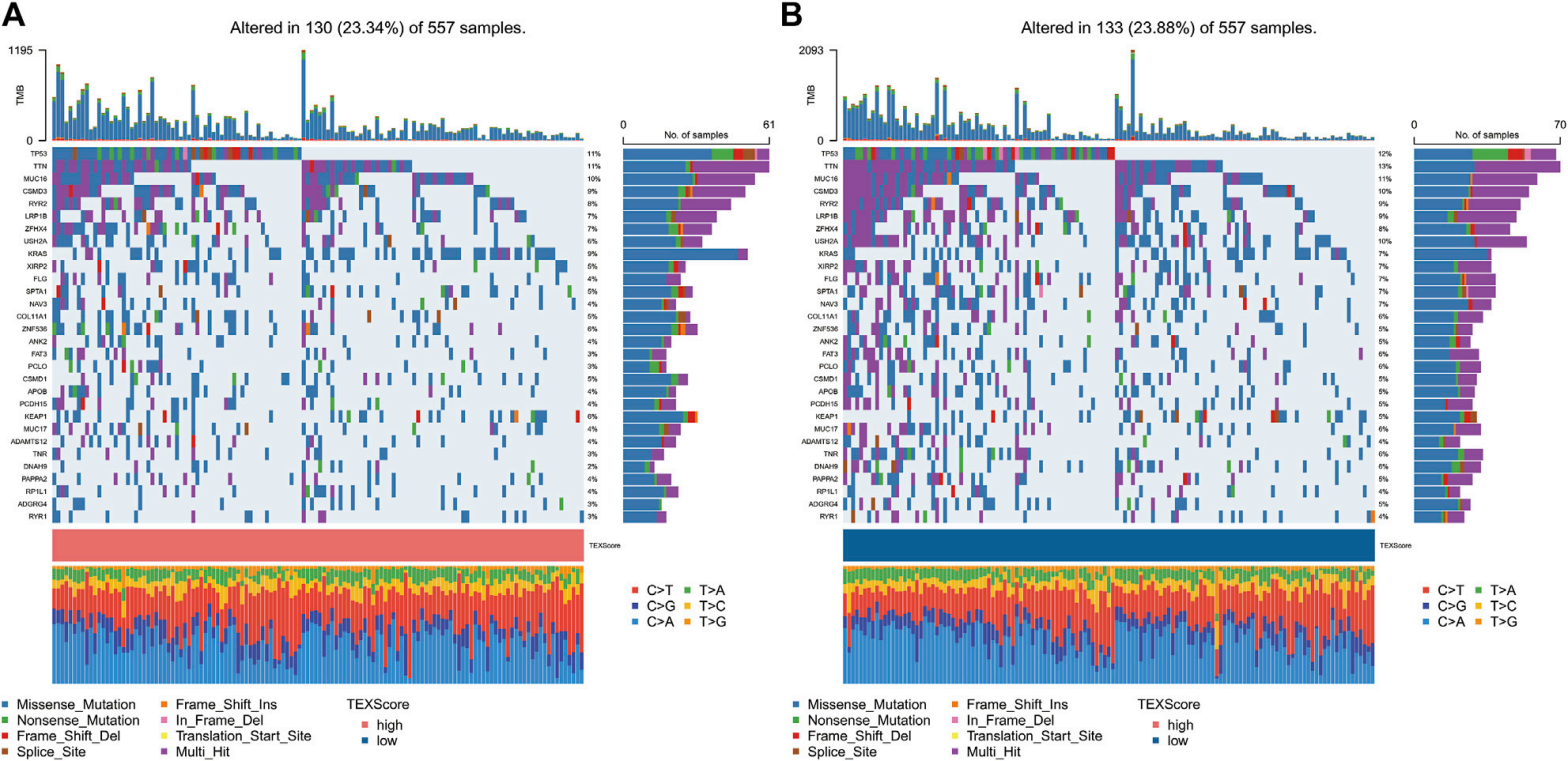
Gene mutation landscape. Gene mutation landscape in high **(A)** and low risk groups **(B)**.

We used a map to determine the landscape of gene mutations in high and low-risk subgroups of patients (Figures 6A, B). There was no statistically significant difference between the gene mutation frequencies between the groups that were analyzed, but it was noted that TP53 and TTN had the highest mutation frequencies.

### 3.8 Relationship between TEX risk score and immunotherapy

**FIGURE 7:**
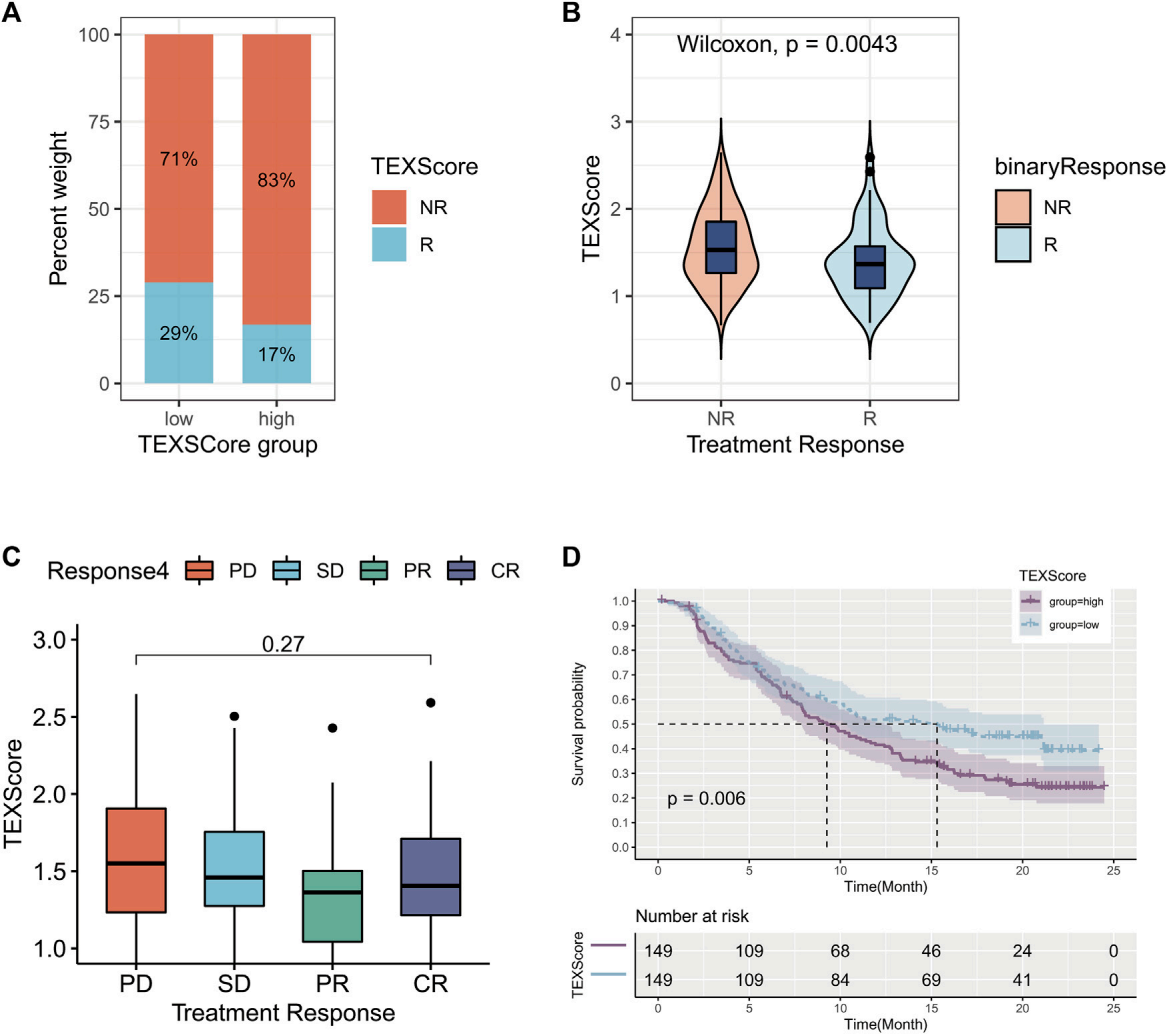
Relationship between TEX risk score and immunotherapy. Percentage weight with response (R) and no response (NR) in the high and low risk groups **(A)**. Wilcoxon test for TEX scores in response and no response populations **(B)**. Boxplots of TEX scores in the four treatment responses **(C)**. K-M survival curves for the high and low risk groups in the immunoresponsive population **(D)**. PR, partial response; CR:complete response; PD: progressive disease; SD: stable disease.

Based on the TCGA-LUAD data set and the IMvigor210 data set, subgraph analyses were conducted to evaluate immunotherapy and chemotherapy in high-risk and low-risk groups. The high-risk group demonstrated a lower percentage of responders to immunotherapy (Figure 7A), and TEXscore was lower in those with low response (Figure 7B). These results suggest that patients with lower TEX risk scores are able to achieve a better immunotherapy response. Specific immunotherapy responses fall into four types: CR: complete response; PR, partial response; SD: stable disease; PD: progressive disease. There was no statistically significant difference in TEX risk scores among the four types of response, suggesting that the specific immunotherapy response was not related to the risk score (Figure 7C).

It was found that the high-risk group had also a poorer overall survival rate than the low-risk group when they received immunotherapy, regardless of the median risk score (Figure 7D). These results suggest that the TEX risk score has a role in predicting the efficacy of immunotherapy and the prognosis of patients receiving immunotherapy.

### 3.9 Single-cell sequencing analysis revealed the therapeutic targets of TEX

**FIGURE 8:**
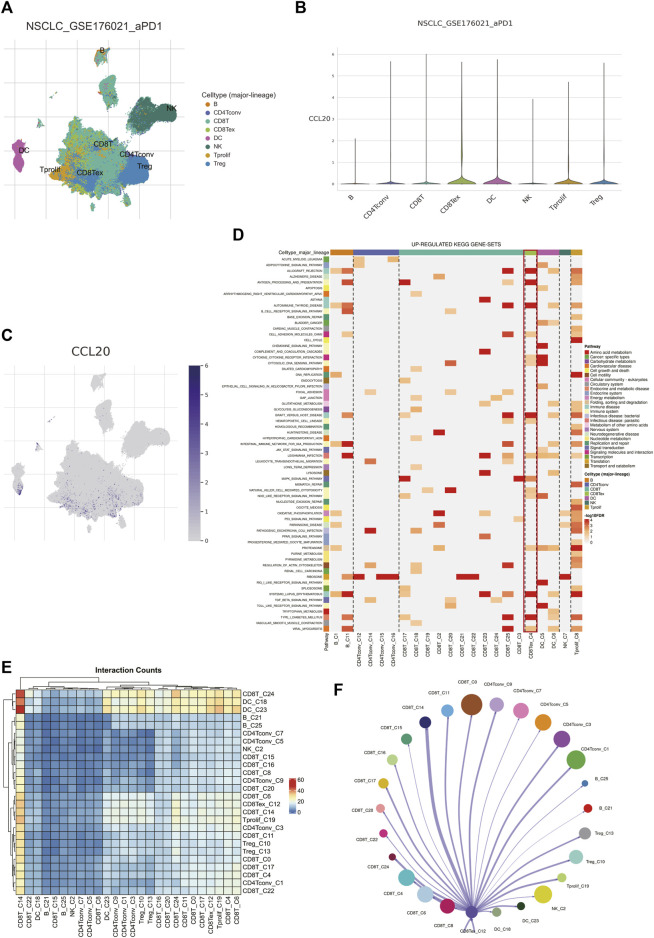
Analysis of TEX by single-cell sequencing. Major subtypes of cells **(A)**. The amount of CCL20 expression on different cells **(B,C)**. Up-regulated kegg pathways in different cell types **(D)**. Interaction conunts of different cell subtypes **(E)**. Diagram of the interaction network between TEX and other cell types, with the width of the network edge being the total number of ligand and receptor pairs **(F)**.

To further search for the potential therapeutic target-cell interactions of TEX, a total of 8 cell subtypes were identified in the single-cell sequencing dataset of T cells (B, CD4 Tconv, CD8T cell, CD8Tex, DC, NK, T prolif, Treg) (Figure 8A). The key gene in the risk model, *CCL20*, was most highly expressed on TEX cells (Figures 8B,C), *CCL20*, a key gene in the risk model, was most highly expressed on TEX cells, suggesting that *CCL20* plays an important role in the TEX process in LUAD patients and is a potential therapeutic target. GSEA analysis showed that TEX cells were mainly enriched in cell adhesion (Figure 8D). The results of cell communication showed that TEX mainly interacted with CD8T cells (Figures 8E,F). These results provide new explanatory theories and therapeutic targets for TEX depletion in LUAD.

### 3.10 GEPIA2, real-time quantitative PCR, and flow cytometry validation

**FIGURE 9:**
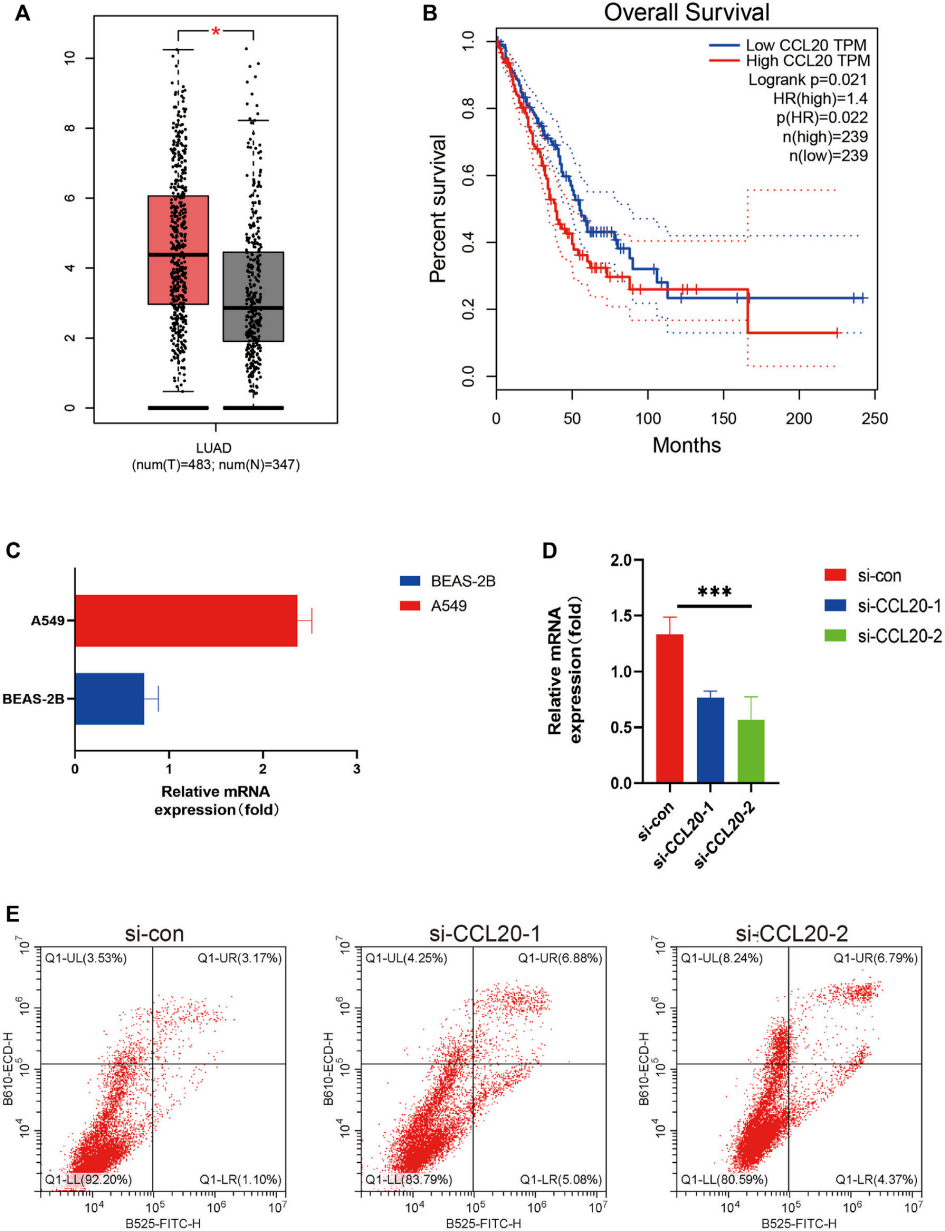
GEPIA2, Real-time quantitative PCR, and flow cytometry validation. *CCL20* expression in LUAD tissues and normal lung tissues in GEPIA **(A)**. K-M curves of overall survival of LUAD patients with high and low *CCL20* expression in GEPIA2 **(B)**. Relative mRNA expression of *CCL20* in A549 cell line and BEAS-2B cell line **(C)**. After knocking down *CCL20* in A549 cells by siRNA, the expression of *CCL20* gene in the three groups of cells was detected **(D)**. The number of apoptotic cells in the three groups was counted by flow cytometry **(E)**. * <0.05.

The GEPIA2 website contained 483LUAD patients and 347 normal lung tissues, and we found the expression level of *CCL20* was higher in tumors tissues. Subsequently, the LUAD patients were classified into diverse subgroups based on *CCL20* expression value (Figure 9A), and the results also showed that the high expression group had a shorter overall survival (*p* = 0.022) (Figure 9B). In addition, we detected *CCL20* mRNA values in both normal and tumor cell lines. The results displayed that the mRNA expression level of *CCL20* in A549 was more than twice that in BEAS-2B (Figure 9C). Subsequently, we knocked down *CCL20* in A549 cells by siRNA (Figure 9D), and the *CCL20* knockdown cells had more apoptosis than the control cells (Figure 9E).

## 4 Discussion

A growing body of evidence suggests that TEX is the result of delayed phenotypic differentiation as well as intermediate functional stages within T cells that follow a sustained state of hierarchy dysfunction. Like other forms of cellular differentiation, it is believed that TEX is the result of T cell hierarchical dysfunction over a prolonged period of time (Wherry, 2011; Jiang et al., 2015; Blank et al., 2019). By understanding CD8 + T cell dysregulation and exhaustion in the tumor microenvironment (TIME), we can overcome the TEX barrier and improve immune checkpoint blockade therapies in the clinic, regardless of whether the type of tumor is the same or different (Kurtulus et al., 2019). The dynamics and heterogeneity of TEX in the TIME are not well studied across LUAD.

In this study, we preferred unsupervised CLUSTER analysis of 284 LUAD patients based on the three most closely TEX signaling pathways (IFNG, TNF, and IL-2), and the patients were divided into four clusters. In cluster D, the number of LUAD patients with the lowest signal pathway score and the largest number of stage1 was the highest. The K-M curve showed that cluster D patients had a better prognosis. PD-1 overexpression leads to inhibitory signaling and induces TEX, leading to tumor immune escape (Zwergel et al., 2022), which suggested that we can use TEX related pathways for prognosis judgment and precise treatment of LUAD. Analysis of immune cell infiltration in four TEXclusters 22 by cibersortx revealed that higher abundance of mast cells resting in TEXa and TEXc was associated with worse prognosis. The existence of mast cells is associated with the prognosis of patients with lung adenocarcinoma, as exosomes derived from mast cells have been shown to promote the proliferation of lung adenocarcinoma cells (Xiao et al., 2014; Bao et al., 2020). However, the specific mechanism of TEX and mast cells needs to be further studied.

WGCNA analysis of the differentially expressed genes and survival random forest analysis obtained 7 key genes (*SOX9*, *CD109*, *CCL20*, *DUSP5*, *DKK1*, *TNS4*, *and LCAL1*). Then we selected 5 genes (*CD109*, *CCL20*, *DKK1*, *TNS4 and TRIM29*) by lasso regression algorism to build a TEX risk model. In the training set TCGA-LUAD and the validation set GSE, high-risk patients had worse overall survival. The AUC value and multivariate cox regression analysis of TEX risk model in training set and validation set showed that Tex risk model had good predictive value and clinical application value. Cluster of differentiation 109 (*CD109*) is a glycosylphosphatidylinositol-anchored protein (Lee et al., 2020). Further studies showed that *CD109* promoted lung adenocarcinoma invasion and metastasis *in vivo* through TGF-β signaling pathway (Chuang et al., 2017; Lee et al., 2020; Taki et al., 2020). However, there is no study on *CD109* and TEX.

Through the enrichment analysis of GSEA and GSVA, we found that TEX score was positively correlated with glycosaminoglycan degradation, linoleic acid metabolism, o glycan biosynthesis, leukocyte transendothelial migration, focal adhesion, ECM receptor interaction and P53 signaling pathway. Targeting P53 has been shown to restore CD8 + T cells depleted in hepatitis C virus infection. However, other pathways and TEX pathways are still worthy of further exploration in LUAD. Subsequent immune checkpoint analysis revealed that the high risk group had increased expression of *CD44*, a stemness marker of non-small cell lung cancer, and activation of *CD44* related pathways promoted squamous cell lung cancer resistance to FGFR1 inhibition (Elakad et al., 2022; Panda and Biswal, 2022). These results suggest that TEX may be involved in the stemness and other phenotypes of LUAD resulting in a poorer prognosis in high-risk patients.

Furthermore, in the pathway enrichment analysis, we found that bile acid metabolism, peroxisome, and T cell receptor signaling pathways were significantly enriched. It has been shown that bile acids can regulate cell growth and proliferation and that alterations in bile acid levels in disease states are associated with liver injury/regeneration and tumorigenesis (Li and Apte, 2015). Peroxisomes can regulate various biological processes and play an important role in several diseases and conditions, and some studies suggest that they may also have an important role in the development and progression of cancer and may represent a new opportunity for cancer therapy (Peters et al., 2005; Youssef and Badr, 2011). In contrast, T cell receptor-based immunotherapy has been shown to be a promising approach for the treatment of various types of cancer. TCRs can recognize epitopes of proteins from any subcellular compartment, including the membrane, cytoplasm and nucleus, and these advantages allow TCRs to detect a wide range of targets, such as neoantigens, cancer germline antigens and viral oncoproteins, and in the clinical setting TCR-based immunotherapy can mediate solid regression of malignant tumors, including immune checkpoint inhibitor-refractory cancers (Schmitt et al., 2009; Kirsch et al., 2015; Chandran and Klebanoff, 2019).

To accurately describe the mechanism of TEX at the single-cell level, we found that the major cells could be divided into eight types through single-cell sequencing data GSE of T cells. It has been found that C-C motif chemokine ligand 20 (*CCL20*) is involved in the occurrence and development of various types of cancer. We found that CCL20, a key gene in the TEX risk score, was highly expressed on TEX cells. In LUAD patients, high expression of *CCL20* is related to epithelial-mesenchymal transition (EMT), which is associated with poor prognosis. Patients responding to anti-PD-L1 therapy were significantly better when *CCL20* expression was low rather than high (Fan et al., 2022). Notably, TNF signaling is also a key pathway in TEX, suggesting that targeting *CCL20* in TEX may have potential clinical value. KEGG signaling pathway analysis identified multiple gene sets up-regulated in TEX, and three signaling pathways attracted our attention. The first is the antigen processing and presentation pathway. Previous findings suggested that patients with higher TEX risk scores had a higher proportion of dendritic cells. The single-cell analysis here further confirms the possible interaction between TEX and DC. Studies have shown that immune checkpoint therapy can restore the immune function of TEX, but it depends on the depleted precursor state of T cells. Dendritic cells provide an important niche for TPEX and prevent its excessive activation (Dähling et al., 2022). Cell communication shows that TEX mainly interacts with CD8T cells, CD8 + T cells differentiate and deplete to TEX, and TEX further acts on CD8 + T cells. This suggests that if we can stop this process, it may provide new ideas for immunotherapy. Subsequent GEPIA2 data analysis, RT-PCR and flow cytometry results similarly indicated *CCL20* as a prognostic indicator for LUAD.

Clinically, there are a number of available risk models based on multiple genes that can predict the prognosis of cancer patients. For example, 21 gene expression analysis (Oncotype DX, Genomic Health) is one of several commercially available gene expression assays that provide prognostic information in hormone receptor-positive breast cancer (Sparano et al., 2018). In clinical practice guidelines for breast cancer, the National Comprehensive Cancer Network (NCCN) strongly recommends 21-gene expression testing (Sparano et al., 2018). Our study now consists of 5 genes and represents a clinically convenient test. Moreover, our model is based on TEX-related genes, which means that our model also has unique potential for predicting immune function in patients.

However, our experiments still have some limitations. Our model performs well, but additional experiments are needed to further validate our model. In addition, although basic experiments were performed to validate one gene in the model, the specific mechanism by which it exerts its function still needs to be explored clearly.

Compared with other traditional models, our model still has great advantages. Our model has not only been validated using different datasets, but also an in-depth analysis based on single cell sequencing data, which will greatly affirm the reliability of our model. Our model can well predict the prognosis and immune control of LUAD patients and provide help for individual precision treatment.

## 5 Conclusion

We comprehensively described the prognostic significance, immunotherapy value and possible mechanism of TEX in LUAD patients for the first time. Nevertheless, the study has certain limitations. Firstly, we defined TEX only according to the scores of three TEX-related signaling pathways, which may simplify the definition of TEX. Secondly, we used public data to analyze the relationship between TEX and LUAD, and there is a lack of molecular biology experiments and *in vivo* results to further confirm our conclusion. In conclusion, our results provide a new insight into the role of TEX in LUAD.

## Data Availability

The datasets presented in this study can be found in online repositories. The names of the repository/repositories and accession number(s) can be found in the article/Supplementary Material.

## Supplementary material

SUPPLEMENTARY FIGURE S1 K-M survival curves for TEXB and TEXD.

Supplementary TABLE S1 Thirty-six genes in the Grey module in WGCNA.
