# Supplementary figures and images for: A systematic and comprehensive analysis of T cell exhaustion related to therapy in lung adenocarcinoma tumor microenvironment
Source: Front Pharmacol. 2023 Feb 6;14:1126916. doi: 10.3389/fphar.2023.1126916 (PMC9939659; doi:10.3389/fphar.2023.1126916)

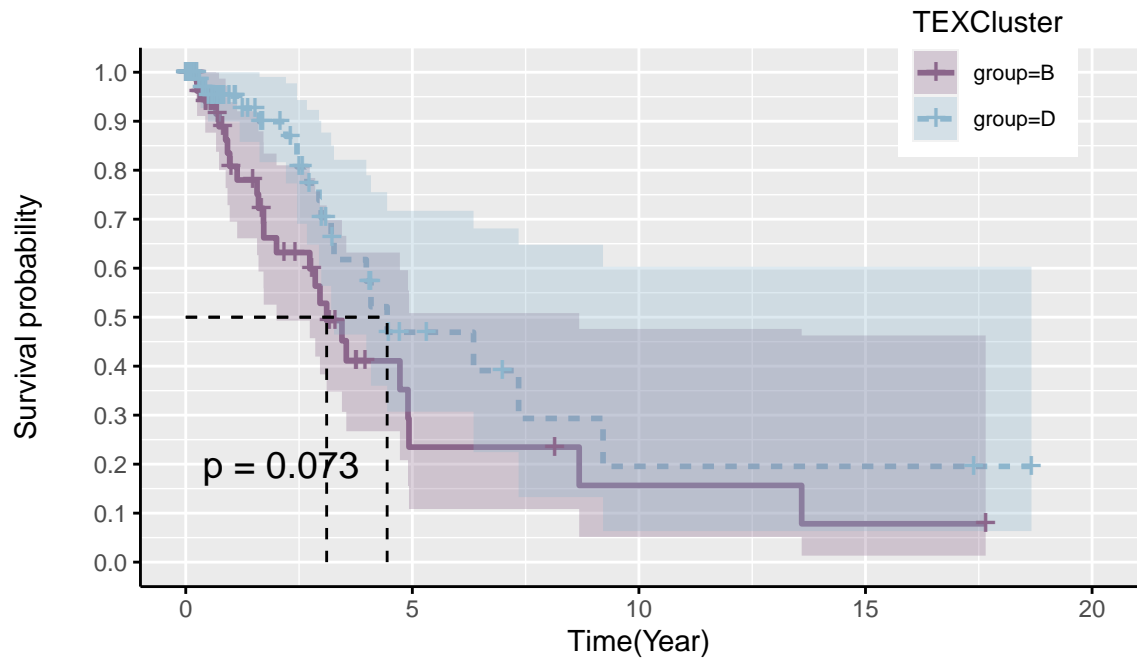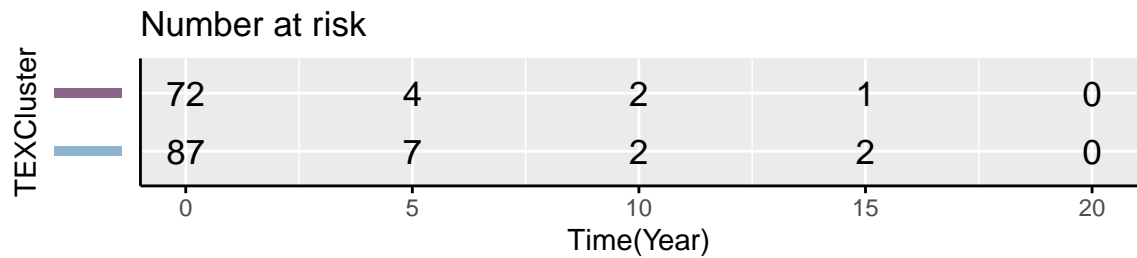

Supplement: Supplementary file 1 [file DataSheet1.PDF]
